# Clinical Audit of Psychiatrist Reviews of Patients on Depot Antipsychotic Medication Under a General Adult Community Mental Health Service in North Norfolk

**DOI:** 10.1192/bjo.2024.613

**Published:** 2024-08-01

**Authors:** Oluseyi Olajide

**Affiliations:** Norfolk and Suffolk Foundation Trust, Norwich, United Kingdom

## Abstract

**Aims:**

In outpatient settings, depot administrations are done to a large extent by Community mental health nurses and other trained clinical personnel who are not psychiatrists. As a result of this, there is a possibility that patients who are having depot medications are not reviewed by a psychiatrist for a long duration of time which can be more than a year. The aim of this audit is to find out if patients currently taking depot medication under a General Adult Community Mental Health Service in North Norfolk are being reviewed by a psychiatrist according to the standard guidelines. The Maudsley prescribing guidelines states that all patients receiving long-term treatment with antipsychotics medication should be seen by their responsible psychiatrist at least once a year (ideally more frequently) to review their treatment and progress.

**Methods:**

List of patients currently on depot medication were taken from the spreadsheet on the Unit's Shared drive. All 47 patients currently receiving depot medication on this list were reviewed. The review period was from Ist January 2023 to 31st December 2023. Psychiatrist Review included Reviews/Appointments by Consultant Psychiatrists, Specialty registrars, Trainee doctors and GPST doctors in psychiatry posting.

**Results:**

It was recorded that 28 patients on depot medication have been reviewed within the last one year which is approximately 60% of the patients currently on depot medication. 19 patients who are currently on depot medication have not been reviewed by a psychiatrist in the last one year, which is approximately 40% of the patients on depot medication. Out of the 19 patients who have not been reviewed in the last one year by a psychiatrist, only 8 of them were offered an appointment.

**Conclusion:**

We can conclude only 60% of patients currently on depot medication were seen by a psychiatrist for a medication review in the last one year. This fell below the expected target of having 100% of these patients meeting with the standard that states all patients receiving long-term treatment with antipsychotic medication should be seen by their responsible psychiatrist at least once a year. A significant proportion of patients might have been deprived of an adequate assessment of their progress and response to treatment and the review of the side effects of these depot medications. These findings have been discussed with the Community Team Manager who has agreed to facilitate that these patients are reviewed promptly.

